# Cultural Historical Psychology and the Reset of History

**DOI:** 10.1007/s12124-021-09649-1

**Published:** 2021-10-09

**Authors:** Pablo del Río, Amelia Álvarez

**Affiliations:** 1 Fundación Infancia y Aprendizaje, Madrid, Spain; 2 Department of Communication, Universidad Carlos III, Madrid, Spain

**Keywords:** Vygotsky’s Legacy, Higher Psychological Functions Changes, Neuroplasticity, Search of meaning, Transhumanism

## Abstract

The authors argue that, in the research trajectory of cultural historical psychology, there are nuclear aspects of Vygotsky’s theory that have been insufficiently considered. Three of these aspects are herein discussed: the intense and rapid changes to mediational processes and their influence on human psyche; meaningful findings on neuroplasticity that require a neuropsychological approach; and, perhaps most importantly, the need for cultural historical approach, and for psychology at large, to return to the study of the direction and meaning of human life.

For several decades, scholars from all around the world have invested a considerable and sustained effort to recover Vygotsky’s legacy, allowing his work to become a reference point in contemporary psychology. Among the works revisited in this process are the ones commemorated in this volume. They have enabled us to access crucial aspects of the historical context in which Vygotsky’s thought developed (Van der Veer & Valsiner, 1991), and the no less important, and not very well-known until now, aspects of Vygotsky’s personal drama, the engine that propelled his scientific and existencial quest (Zavershneva & van der Veer, 2018b). The dramatic aspect and the search for meaning in Vygotsky’s thinking has always been the central axis of our approach to his work; we thus celebrate Zavershneva and van der Veer’s recent work on Vygotsky’s unpublished personal writings twice over.

## Cultural-Historical Psychology Problems, One Century Later

### A History of Changes

It is well-known that the theory proposed by Vygotsky was a system that situated the genesis of higher psychological functions in the action of culture, via mediations, throughout history. Thus, the cultural-historical denomination he himself coined places two points of emphasis, from which we wish to frame this commentary. On one hand, the cultural emphasis underlines that it is not just biology, but also culture, that which determines the construction of higher psychological functions; on the other hand, the historical emphasis underlines that higher psychological functions are a product of history, both that of the species’ (phylogenesis) and of each individual of that species (ontogenesis) (Veresov, 2020, p. 111). Thus, it can be concluded that changes in human history, and in each personal biography, will continuously transform our science’s object (del Río & Álvarez, 1995).

In our opinion, and in line with Matusov’s criticism (2008), the cultural historical school should have dedicated more research to these tenets of the Vygotskyan theoretical system. This deficit is all the more poignant because almost 90 years have gone by since Vygotsky’s passing, almost a century of cultural historical changes —perhaps the rapidest, most accelerated changes humanity has seen throughout all its history. If history and culture change us, how have they changed us after all this time?

Other disciplines, within psychology and other sciences, have studied and alerted about these changes. For instance, media psychology, media ecology, and communication studies have contributed ample research on the influence of mediational change in the development of different psychological functions, such as attention, perception, memory, thinking, emotion, direction of behavior… every higher function is being transformed continuously (Carr, 2020; del Río, Álvarez & del Río, 2004; Pea & Cole, 2019).

This perceptive lacuna of cultural historical psychology is worrisome: not paying attention to cultural change does not precisely help when preparing to face it. We will come back to this below.

### Mediation’s Heads and Tails

At the onset of biocybernetics, Heinz von Föerster suggested that, to be able to upgrade into pluricellular organisms, unicellular organisms, which until then held a direct relationship with the outer world, had to deal with the problem of recognizing, reconstructing, and defining reality —and they did it through complex celular systems of tiered inner representation which put them in indirect contact with the outer world: recurring computing or second-order cybernetics (von Foerster, 1974).

The human being pictured by Vygotsky is an organism that transcends its body limits to, through culture, extend its psychic functions and free itself from the spatial-temporal limitations that keep animals tied to their present, and to what is present.

But constructing reality through this new, external mediation process, entails a systemic redefinition of the organism and of the environment. In the new environment, the human organism inserts in the preexisting natural medium (the basic, animal ecology) a second ecology of external organs and affordances. These external mediations (extracortical connections) must be somehow integrated with the preexisting internal mediation system, thus creating a new organism.

While the first, natural process analyzed by von Foerster took place for over two billion years, the externally mediated reconstruction of reality (culture) is comparatively very recent: to use Carl Sagan’s cosmic annual calendar analogy (January 1st would be the creation of the Universe, 15 billion years ago), we have been experiencing it only for the two hours just before the new year. It is thus to be expected that the adaptation to this new, game-changing process would not take place without difficulties —the novel mechanism of cultural mediation not only demands a radical reconstruction of reality, it also entails that the new, intermediate constructions, while indeed useful, have to be regarded as symbolic and unreal. The integration will therefore be troublesome, and human beings will tend to move in a potentially schizoid territory:

each of us carries his own latent schizophrenia with him (…) Advertising also guides our actions; its relation to action is not the sad privilege of schizophrenia (…) Advertising convinces us to buy what we do not need. (Zavershneva & van der Veer, 2018b, p. 321).

As we have pointed out elsewhere, the apparition of technologies that increasingly supplant original referents greatly expands the structural difficulty inherent to the human species’ mediational process, rendering the process of constructing reality (and of dealing with irreality) for new generations much more challenging (del Río & Fuertes, 2004). Indeed, the problem of constructing reality marks the ambivalent and flexible nature of the cultural construction of higher functions. The challenge for human development is that each new cultural architecture determines a functional architecture that not only provides new potentialities but, to use McLuhan’s term (1964), new “amputations” of previous systems, new areas of “apoptosis”, and new risks of pathologies and functional disorders. We believe that monitoring the changes in cultural architectures and their funcional correlates as they emerge should be a central task for cultural historical psychology.

### A Restless Brain

What is the connection between internal mediations analyzed by Von Foerster’s biocybernetics and external mediations proposed by Vygotsky? As mentioned, external mediation is a relatively recent process in evolution, and it must be integrated with a biological system of a much longer evolutionary history. In his final years, Vygotsky was not at peace with his model of “internalization”, as he thought it was a psychological but not yet biological concept; an idea one step away from dualism. He thus proposed that higher psychological functions do not simply supplant natural functions, but *restructure* them, and that this process must be reflected in cortical development.

Thus, his first investigations about brain organization, made from the medical field, and that Luria would later continue, led him to an inevitable conclusion: the brain must change to integrate extracortical connections, which necessarily implies cortical plasticity (del Río & Álvarez, 2017). Although Luria’s research and that of his colleagues eventually presented with evidence of cortical changes accompanying the development of higher functions (Fárber, 1978/1983), and even though Luria’s work has come to be well-known and respected in the West, Vygotsky’s theory of brain plasticity was somewhat forgotten. Perhaps it was difficult to integrate in a neurological science that, since Ramón y Cajal coined the term “neuroplasticity”, had upheld a rather static view of the brain, a perspective that prevailed practically all throughout the Twentieth Century. But in the last three decades, and from very different fronts, research on neurogenesis has found ample evidence for neuroplasticity, both in synaptic processes as well as in the genesis of new neurons (Doidge, 2007; Merzenich, 2013).

Even if neuroscience has found evidence that the brain changes, it still lacks a psychological theory that explains why; Vygotskyan psychology offers a promising way for the development of precisely such a theory. And with regards to cultural historical approach itself, overlooking the path opened by Vygotsky’s and Luria’s neuropsychology would be incongruent: if, as Toomela (2016) has underlined, cultural historical psychology has no choice but to be a neuropsychology, it should not limit its scope to the clinical setting, but rather guide functional development in every domain of human life (Akhutina & Pylaeva, 2008).

All these realities, historical genesis, cultural genesis, neurogenesis, and even the recent recovery of the concept of epigenesis (Carey, 2011; Gottlieb, 2007) support Vygotsky’s theory, and indicate that, in order to continue its theoretical development, Vygotskyan psychology needs a cross-discipline expansion; and, in order to orient its empirical research, it must account for changes and interactions between a culture that changes —and a brain that falls suit.

## The Silent Search for Meaning

The stone the builders rejected has become the cornerstone (Psalm 118:22)

(Vygotsky, 1927/1982/1991, p. 259)

### The Road to Freedom

The era of cognitive psychology’s predominance was marked by its capacity for propositional and informational analysis, and its productivity in generating technologies and industries. But all focusing creates periphery, all emphasis on a given area relegates other areas and thus, by omission, mainstream psychology has created a shadowed area, leaving out or entirely forgetting the processes that organize and direct human behavior.

As we have proposed elsewhere (del Río & Álvarez, 2007a, b, c, 2017), Vygotsky showed from start to finish a special interest for processes that motivate and direct people’s lives: emotion, drama, will, and freedom. This interest is explicitly mentioned in *Psychology of Art*, and it never declined —it permeates and upholds all his work, converging finally in his psychology of heights: the conquest of freedom. Along with the re-founding of psychology and of research on conscience, a second, more personal quest seems to have closely escorted Vygotsky: the search for meaning, for destiny. That is the cornerstone that upheld his whole psychology of heights.

Thus, Zavershneva and van der Veer (2018a), when revising Vygotsky’s writings between 1914 and 1917, point at the mystic and religious thirst he pitted against more basic social goals such as primary needs (man does not live on bread alone). In his first works on psychology, this spiritual impetus of his youth re-focuses on the social and scientific revolution. In his late personal notes, the focus on personal ascension, an almost invisible companion in the background of his mid-life work, re-emerges to the forefront, in an attempt to articulate it with his better-known ideals of social transformation.

After a quest for the mediational and neuropsychological architecture of conscience, Vygotsky thinks to have discerned a conscience capable not only of handling meanings, but of capturing the ultimate meaning of things and, from it, to elevate towards freedom: his acmeist, “height” psychology. He then also ponders on a methodology (semic analysis) capable of researching this new field of conscience oriented to meaning.

In his last stage, Vygotsky elevates the whole directive process to an almost utopian level. Personal construction emerges as a quasi heroic adventure, like a difficult ascension towards freedom:

The grandiose picture of personality development: the path to freedom. Only here does psychology as a science begin. (Vygotsky, in Zavershneva & van der Veer, 2018b, p. 209).

According to the laws of nature, man is not a free being: People are not born free. (…) Freedom is not given; it is taken (…) Man can become free, but this is as excellent as it is rare (…) It is not located in the depths but in the summits of the mind (…) in freedom that became life (a way of life) (…) The *Amor Dei intellectualis* leads to the highest manifestation of human nature, to the power of the spirit (wisdom is more powerful than hope), to the activity of the autonomous free person. In essence, this is the *amor fati*: the love of fate. (Vygotsky, in Zavershneva & van der Veer, 2018b, p. 374).

Vygotsky is here proposing a challenging program, limited maybe only to the most zealous, to those devoid of vertigo and fear of heights. Everything seems to indicate that life leads most human beings through a path oscillating between presentiality and future planes, between everyday affairs and the most elevated ideals. But can Vygotskyan theory offer a psychology of conscience to the common man? A psychology of heights in which summits, if not pervasive, can be reachable to all? This is a key point, because on it depends whether the utopia of personal destiny and the utopia of social improvement can be articulated.

It is a pending task, but at least it’s no longer a solitary adventure. Significant areas of science are blooming with proposals which seem to recover intentionality as inherent to the processes of life. Thus, after decades of preeminence of theoretical models whose postulates are based on chance and competition, different proposals showing this re-orientation have appeared, highlighting ideas such as:

- Direction and intentionality in biological processes: a new look at evolution questioning pure chance. *Autopoiesis* (Maturana & Varela, 1980) or *lifelines* (Rose, 2005a) are relevant concepts in this perspective.
- Symbiosis and cooperation among living beings: processes that intervene in evolution, displacing the idea of the struggle for life as the sole, governing principle (Lane, 2009; Margulis & Sagan, 1997).
- Integration organism-environment: the organism is a part of the environment, and the environment is a part of the organism; the process of epigenesis mentioned above is framed in this reorientation.

Intentionality was banished from science and shown the door—but it seems to be creeping in through the windows. Now, the impermanent and elusive side of matter is being sought, as are plasticity and the brain’s ability to capture more ample records of reality; less evident vectors or factors behind the process of evolution are being sought too. It is still early to predict where this transition will lead throughout this century, but after the *Notebooks* publication, we believe that Vygostky’s legacy is now more explicit, and it opens a stimulating field of research.

### The End of History—again?

In the final pages of *The Historical Meaning of the Crisis in Psychology,* Vygotsky (1927/ 1982/1991) expressed his faith in that the key product of history, conscience, turned upon itself, would be able to produce a recreation of human beings, with the participation of biology and psychology. Psychology would be central because for the new human being the command of his own being will be also central, his own subordination to himself: freedom. The publication of these annotations confirms this teleological idea, even though it shifted gradually, did not abandon Vygotsky’s thought since he first formulated it.

On the other hand, the aspiration to re-create human beings is a driving idea that has repeated itself throughout history. In this respect, we sense a clear parallelism between the 20s of the Twentieth century, the time in which Vygostky attempted his project of re-foundation of psychology, and the current 20s of the Twenty-first century, when those psychologists based on his work try to lay out our own psychology. After all this time, do we still share our object of study? Is psychology’s subject the same after a century?

For human re-creation has take two very different paths: in Vygotsky’s time, the humanist ideal was still pursued; today, an alternative ideal has emerged (transhumanism), fiercely disputing, out of the gates, the right to define what to be human means.

A clear example of this dispute can be found in these two citations which, a hundred years apart, formulate two very different ideals of human re-creation:

In this sense Pavlov is right when he calls our science the last science about man himself. It will indeed be the last science in the historical or prehistorical period of mankind. The new society will create the new man. When one mentions the remolding of man as an indisputable trait of the new mankind and the artificial creation of a new biological type, then this will be the only and first species in biology which *will create itself* … (Vygotsky, 1927/1982/1991, p. 406, our emphasis)

“(…) once technology enables us to re-engineer human minds, Homo sapiens will disappear, human history will come to an end and a completely new kind of process will begin” [humanity will have then] “divine powers of creation and destruction, and upgrade Homo sapiens into Homo deus” (…) “The upgrading of humans into gods may follow any of three paths: biological engineering, cyborg engineering and the engineering of non-organic beings” (…) (Harari, 2016, Chapter 1, Kindle Edition)

1. Science is converging on an all-encompassing dogma, which says that organisms are algorithms and life is data processing.

2. Intelligence is decoupling from consciousness.

3. Non-conscious but highly intelligent algorithms may soon know us better than we know ourselves.

(Harari, 2016, Chapter 11, Kindle Edition)

The assumptions that Harari perceives in current science and society are, *grosso modo*, those that are supporting both the so-called “Fourth Industrial Revolution” (Schwab & Malleret, 2020), already underway, as well as the model of human being called “transhumanism” by its instigators. A model of human being very distant from that proposed by Vygotsky, in which the construction of the new man was conceived as coming from conscience and freedom.

What the final result of these experiments of the new engineerings that aim to control our soma and our psyche will be, or whether it is possible for intelligence to decouple from conscience, only experience and empiric research will eventually tell us. Whether intelligent algorithms may “know” us — and control us better than we ourselves can.

In the meantime, we sense several problems in the approach to the redesign of biological and psychological processes that is currently being applied, although here we will only point out two of them.

On the one hand, both from a Vygostkyan perspective as well as from holistic biology and psychology, intellectual functions operate integrally with directive functions, so that a non-directive intelligence wouldn’t be very intelligent.

Which leads us to another problematic assumption being proposed by the AI industry and its research pundits: that the human directive system can be hacked and replaced by an artificial operative system.

Vygotsky had already pointed out that cultural mediations enable human beings to build cultural organs in the outer world, trans-organic (del Río & Álvarez, 2017). Currently, the development of computing and biological engineering enables these exo-organs to be implanted as intra-organs, creating a problem of neuropsychological integration.

In this vein, when pondering about the innovation behind the combination of neuroscience and computing (ciborgery), Steven Rose (2005a, b) alerted about a potential denaturalization of the brain, of biology and of psychology, if these exo-organs are made internal.

Let us remember that, according to the laws of neuronal growth proposed by Pribram or Luria, cortical development is produced in natural situations by accretion, by progressive and tiered integration of new cortical connections that are added to the pre-existing ones, safeguarding the integrality, hierarchy and base dynamics of the system. This entails that, in the same way that internal neurogeneses springing from the use of a new psychotechnical extension do not take over the control of conscience but, on the contrary, conscience takes control of them, the exo-organs, introjected inside the organism, should not be imposed upon the central circuits of intra-organic conscience. According to Bacon’s law (*natura parendo vincitur*), Vygotsky argues that mediated, functional reconstruction of higher functions following the laws of the natural brain, as has taken place to date in historical genesis, safeguards the fluidity and integrality of human development.

We are facing a historical challenge, one that takes us back to the confrontation between systems and fate mentioned by Vygotsky:

The living, real, *individual person and his destiny forms the fundamental problem of psychology* (Zavershneva & van der Veer, 2108b, p. 222, our emphasis). When we pass from systems to fates (pronouncing this word is terrifying and joyful at the same time, knowing that tomorrow we will investigate what is hidden behind it), to the birth and downfall of systems, we see it with our own eyes (letter to his student Levina, 16 July 1931, Vygotsky, cited by van der Veer & Valsiner, 1991, p. 16).

At any rate, we cannot now evade neither systems nor fate. Psychology, whether it wants to or not, must updated its object of study, for human history has reached a point in which mankind can assume its future and, for better or for worse, redesign itself. It *can*, but does it *know how*? How, for what, and towards what — the meaning of such recreation, the “chosen destiny” — is of vital importance for all human beings and for the planet they inhabit, and it should be the central point of a clear and ample (and, of course, scientific) public debate. Cultural historical psychology should have an active participation in this debate.

*Forgive me, dear creatures. The rest is silence.* (Vygotsky, en Zavershneva & van der Veer, 2018b, p. 497).

With this last personal note, Vygotsky bids us farewell, leaving us at the same point as his theoretical writings: at the gates of a promised land, and before the same contradictory and unfinished quest for meaning in psychology. His quest returns to silence, his cornerstone will perhaps become just another stone, shrouded again in mystery, discarded as so many times before.

Was he perhaps begging for pardon from his “dear creatures” for not having completed that quest, a plea for his followers to continue it? We now understand that Zavershneva & van der Veer’s comment in their introduction to the Notebooks (Zavershneva & van der Veer, 2018b, p. xxi) is in reality a challenge: “Where Vygotsky stopped, psychology must move on”. (Zavershneva & van der Veer, 2018b, p. xxi).

## Data Availability

Not applicable.

## Declarations

To be used for all articles, including articles with biological applications.
