# Correction to: Cultural Historical Psychology and the Reset of History

**DOI:** 10.1007/s12124-021-09666-0

**Published:** 2021-11-03

**Authors:** Pablo del Río, Amelia Álvarez

**Affiliations:** 1 Fundación Infancia y Aprendizaje, Madrid, Spain; 2 Department of Communication, Universidad Carlos III, Madrid, Spain

**Correction to** **:** **Integrative Psychological and Behavioral Science**

**https://doi.org/10.1007/s12124-021-09649-1**

The original version of this article contained mistakes.

Under the header "The End of History—again?", p. 7, the paragraph:

What the final result of these experiments of the new engineerings that aim to control our soma and our psyche will be, or whether it is possible for intelligence to decouple from conscience, only experience and empiric research will eventually tell us. Whether intelligent algorithms may “know” us long dashes, please and control us better than we ourselves can.

Should be corrected to:

What the final result of these experiments of the new engineerings that aim to control our soma and our psyche will be, or whether it is possible for intelligence to decouple from conscience, only experience and empiric research will eventually tell us. Whether intelligent algorithms may “know” us — and control us — better than we ourselves can.

Under the same header, p. 8, the paragraph:

At any rate, we cannot now evade neither systems nor fate. Psychology, whether it wants to or not, must updated its object of study, for human history has reached a point in which mankind can assume its future and, for better or for worse, redesign itself. It can, but does it know how? How, for what, and towards what long dashes, please the meaning of such recreation, the “chosen destiny” is of vital importance for all human beings and for the planet they inhabit, and it should be the central point of a clear and ample (and, of course, scientific) public debate. Cultural historical psychology should have an active participation in this debate.

Should be corrected to:

At any rate, we cannot now evade neither systems nor fate. Psychology, whether it wants to or not, must updated its object of study, for human history has reached a point in which mankind can assume its future and, for better or for worse, redesign itself. It can, but does it know how? How, for what, and towards what — the meaning of such recreation, the “chosen destiny” — is of vital importance for all human beings and for the planet they inhabit, and it should be the central point of a clear and ample (and, of course, scientific) public debate. Cultural historical psychology should have an active participation in this debate.

The original article has been corrected.

